# High Proportion of 22q13 Deletions and SHANK3 Mutations in Chinese Patients with Intellectual Disability

**DOI:** 10.1371/journal.pone.0034739

**Published:** 2012-04-11

**Authors:** Xiaohong Gong, Yu-wu Jiang, Xin Zhang, Yu An, Jun Zhang, Ye Wu, Jingmin Wang, Yangfei Sun, Yanyan Liu, Xuewu Gao, Yiping Shen, Xiru Wu, Zilong Qiu, Li Jin, Bai-Lin Wu, Hongyan Wang

**Affiliations:** 1 The State Key Laboratory of Genetic Engineering and MOE Key Laboratory of Contemporary Anthropology, School of Life Sciences, Fudan University, Shanghai, China; 2 Department of Pediatrics, Peking University First Hospital, Beijing, China; 3 Institutes of Biomedical Sciences, Fudan University, Shanghai, China; 4 Children's Hospital of Fudan University, Shanghai, China; 5 Institute and Department of Digestive Diseases, Huashan Hospital, Fudan University, Shanghai, China; 6 Institute of Neuroscience, Shanghai Institute of Biological Sciences, Chinese Academy of Sciences, Shanghai, China; University of Illinois at Chicago, United States of America

## Abstract

Intellectual disability (ID) is a heterogeneous disorder caused by chromosomal abnormalities, monogenic factors and environmental factors. 22q13 deletion syndrome is a genetic disorder characterized by severe ID. Although the frequency of 22q13 deletions in ID is unclear, it is believed to be largely underestimated. To address this issue, we used Affymetrix Human SNP 6.0 array to detect the 22q13 deletions in 234 Chinese unexplained ID patients and 103 controls. After the Quality Control (QC) test of raw data, 22q13 deletions were found in four out of 230 cases (1.7%), while absent in parents of the cases and 101 controls. A review of genome-wide microarray studies in ID was performed and the frequency of 22q13 deletions from the literatures was 0.24%, much lower than our report. The overlapping region shared by all 4 cases encompasses the gene *SHANK3*. A heterozygous *de novo* nonsense mutation Y1015X of *SHANK3* was identified in one ID patient. Cortical neurons were prepared from embryonic mice and were transfected with a control plasmid, shank3 wild-type (WT) or mutant plasmids. Overexpression of the Y1015 mutant in neurons significantly affected neurite outgrowth compared with shank3 WT. These findings suggest that 22q13 deletions may be a more frequent cause for Chinese ID patients than previously thought, and the *SHANK3* gene is involved in the neurite development.

## Introduction

Intellectual disability (ID), commonly referred to as developmental delay (DD), mental retardation (MR) or learning disability, is a developmental disorder characterized by significant impairment of intellectual function and deficiency in two or more adaptive behaviors, with onset before the age of 18 years. Although the prevalence of ID varies with changes in diagnostic criteria, assessment tools, medical services, culture, and social custom, the estimates are between 1 and 3% in the general population, making ID the most frequent cause of severe handicaps in childhood and one of the main reasons for clinical genetic referral and counseling. As an extremely heterogeneous disorder, ID can be caused by a number of chromosomal abnormalities, monogenic factors and environmental factors. Down syndrome, Fragile X syndrome and fetal alcohol syndrome are the most common syndromes associated with ID [1]. Chromosomal abnormalities, such as aneuploidies, rearrangements, and subtelomeric deletions, play an important role in the etiology of ID, accounting for nearly 40% of cases of moderate to severe ID and approximately 10% of mild ID [2]. In a review of 16,673 patients, subtelomeric imbalances were reported in 586 cases (3.5%) [3]. New high-resolution microarray-based genomic profiling technologies have enabled the detection of submicroscopic chromosomal imbalances (microdeletions/microduplications) throughout the genome. Consequently, genotype-phenotype correlation studies have been undertaken to examine the clinical significance of copy number variants (CNVs) in ID. Koolen et al. reviewed 16 genome-wide microarray studies in 1,364 patients with ID, 11.2% had detectable CNVs related to ID [4]. Sagoo et al. [5] and Miller et al. [6] conducted similar systematic reviews and found overall diagnostic yields of 10% and 12.2%, respectively, for pathogenic genomic imbalances in individuals with ID and/or congenital anomalies. Cooper et al. performed a whole genome-wide array CGH study in 15,767 children with ID and 8,329 unaffected adult controls and estimated that 14.2% of ID was caused by CNVs>400 kb [7].

The 22q13 deletion syndrome, also known as Phelan-McDermid Syndrome, is a microdeletion syndrome characterized by severe ID, an absence of speech or a severely expressive speech delay, hypotonia, normal to accelerated growth, and mild dysmorphic features [8]. This syndrome results from the deletion or disruption of the 22qter region. The frequency of the 22q13 deletion in ID is unclear, but it is believed to be largely underestimated because of the lack of clinical recognition of this syndrome and the limitations of routine cytogenetic techniques used for experimental validation. Fluorescent in situ hybridization (FISH), high-resolution chromosomal analysis and microarrays are commonly used to detect this syndrome in the lab, and reported deletion sizes range from 95 Kb to more than 9 Mb. In the minimal critical region, the gene *SHANK3* is a promising candidate of causative genes for ID. SHANK3, predominantly expressed in the cerebral cortex and cerebellum, encodes a scaffolding protein found in excitatory synapses. It contains multiple protein-protein interaction domains and functions as a master organizer of the postsynaptic density (PSD). In an individual that represented the main characteristics of 22q13 deletion syndrome, the *SHANK3* gene was directly disrupted with a balanced translocation t(12;22)(q24.1;q13.3) [9]. Anderlid et al. reported a 100 Kb terminal 22q13 deletion which affected three genes including the disruption of *SHANK3* [10]. Durand et al. firstly sequenced all *SHANK3* exons in autism spectrum disorders (ASDs) and found a frameshift mutation in one ASD individual [11]. Other independent groups found more *de novo* missense mutations and gene deletions of *SHANK3* in ASD, supporting the involvement of *SHANK3* in the etiology of ASD [12], [13]. Hamdan et al. sequenced a large number of functional candidate genes for ID in 95 cases and found a *de novo* splicing mutation of *SHANK3* [14].

To address the frequency of 22q13 deletions in ID, we performed a high-resolution microarray analysis in a cohort of 234 Chinese unexplained ID patients. Four out of 230 cases (1.7%) were identified to have 22q13 deletions, of which the smallest imbalance segment spanned 113 Kb. A heterozygous nonsense mutation, Y1015X, was identified in a single case but was absent in the unaffected parents and 556 controls. The overexpression of *SHANK3* Y1015X plasmid in primary cultured mouse cortical neurons resulted in reduced numbers of neurite nodes and tips and reduced cumulative length compared with neurons transfected with wild-type (WT) *SHANK3* plasmid.

## Materials and Methods

### Ethics Statement

The study was approved by the Ethics Committee of the Health Science Center, Peking University and the Ethics Committee of the School of Life Sciences, Fudan University. Informed, written consent was obtained from the controls, the parents or guardians of the children.

### Subjects

A total of 234 Chinese patients (166 males and 68 females) were recruited from the Department of Pediatrics, Peking University First Hospital, China. The assessment methods and inclusion criteria for the patients have been previously described [15]. Briefly, patients with unexplained ID were defined as those without evidence for any known genetic or acquired disease causing ID after thorough clinical and related lab evaluations. Gesell Developmental Schedules or Wechsler intelligence scale for children was used to assess IQ scores. The patients were excluded if they had the history of poisoning, hypoxia, central nervous system infection, perinatal brain injury and cranial trauma. Laboratory tests to rule out known genetic causes of ID included standard karyotyping, metabolic screening, fragile X testing for male patients, and *MECP2* gene mutation analysis for female patients. All patients fulfilled the DSM-IV criteria for ID. Ninety-five percent of the ID individuals were Han Chinese.

Two cohorts of controls were recruited for this study. They were 103 Han Chinese controls (53 males and 50 females) for the whole genome analysis of CNVs and 556 controls for the sequencing of the *SHANK3* gene.

Genomic DNA was extracted from whole blood according to standard protocols.

### Detection of CNVs

The Affymetrix Human SNP array 6.0 was used and it contains more than 906,600 single nucleotide polymorphisms (SNPs) and more than 946,000 probes for the detection of CNVs [16]. All 234 cases and 103 controls were genotyped using this array according to the manufacturer's protocol. Raw genotyping data were acquired in the form of CEL files using the Affymetrix GeneChip Command Console (AGCC) software.

CNVs were first analyzed using the Affymetrix Genotyping Console 3.0.1 (GTC, Affymetrix) software. Those samples with contrast Quality Control (QC) less than 0.4 were removed from the subsequent CNV analysis. As a result, 230 patients and 101 controls passed the QC test and were included in the further analyses. Average call rates for the cases and controls were 99.54% and 98.28%, respectively. The copy number analysis was performed using the BRLMM-P-Plus algorithm.

Additionally, the raw data were analyzed using three other software packages including DNA-Chip Analyzer (dChip), PennCNV and Birdsuite to validate the CNVs identified by GTC and by each other. Those CNVs that were detected by at least two of the four software packages were considered to be ‘true’ CNVs. Those CNVs characterized to have more than 50% overlap by different software were considered to be the same CNV.

### MLPA confirmation of 22q13 deletion

Positive cases of 22q13 deletion were further confirmed using a custom-designed MLPA assay. Additionally, the parents of these patients were tested using the same MLPA to determine whether the deletion was inherited or *de novo*. For MLPA analysis, commercially available SALSA® MLPA® Reagents kits (MRC-Holland, Amsterdam, Netherlands) were used. Probes specific for *SHANK3* were designed according to the recommended protocol by MRC-Holland. The probe mix included *SHANK3* exons 2, 3, 5, 9, 10, 12, 13, 16, 17, 20 and 22 as well as 4 *PSPC1* probes included for quality control. The MLPA reactions were performed according to the manufacturer's recommendation, using 250 ng of DNA in a 5 µl reaction. Products were separated by capillary electrophoresis on an ABI 3730 Genetic Analyzer (Applied Biosystems, Foster City, CA), and the data were analyzed using GeneMarker v1.85 (SoftGenetics LLC, State College, PA). Threshold ratios for deletion and duplication were set at <0.75 and >1.30, respectively.

### Literature review of 22q13 deletion

A review of genome-wide microarray studies in patients with ID was performed, from which the frequency of 22q13 deletion was addressed. We used both free text and MeSH terms including intellectual disability, mental retardation, developmental delay, and microarray analysis to search the literature available in the PubMed database of the National Center for Biotechnology Information (NCBI) prior to Aug 2011. Included literature was selected according to the following criteria. First, the description of the study population was clear, and the majority of cases in each study were affected with ID. If the samples that were received for experimental testing had various indications including ID, dysmorphic features (DF), or multiple congenital anomalies (MCA), the percentage of each subgroup should be mentioned in the study to gauge whether the majority of the cases were affected with ID. Second, the pre-tested karyotyping results were normal for the patients. Third, the applied microarray method was whole genomic, not targeted, analysis. Finally, pathogenic CNVs identified in each study should be listed in detail so that the number and location of the 22q13 deletion/duplication can be analyzed.

### Sequencing *SHANK3* gene

The reference sequence for *SHANK3* was acquired from the UCSC Genome Browser (NM_001080420). All exons and exon-intron junctions were sequenced in each of the 234 ID cases using an ABI 3730XL sequencer. We were unable to successfully sequence Exon 12 due to its high GC content. The primer sequences used for amplification are available upon request. Each of the 556 matched controls was genotyped using the SNaPshot method.

### Plasmid construction and transfection

Full-length rat Shank3 cDNA (pcGlobin2-HA-rat-shank3) was received as a gift from Dr. Drapeau Pierre. Mutagenesis was performed using the KOD-Plus-Mutagenesis Kit (Toyobo) according to the manufacturer's protocols. The human Y1015X and A921T mutations identified in our samples correspond to residues Y999X and V905T of the rat shank3 protein, respectively. The primers utilized for A921T mutagenesis were: forward 5′-GCCAGAAGTGGGCGATACACCCCGGCCTG-3′ and reverse 5′-CAGGCCGGGGTGTATCGCCCACTTCTGGC-3′. The primers utilized for Y1015X mutagenesis were: forward 5′-GGGCGGCCTTGACTAGAGCTCTGGAGAAGG -3′ and reverse 5′-CCTTCTCCAGAGCTCTAGTCAAGGCCGCCC -3′. The PCR products were digested with restriction enzymes AseI and ClaI and sub-cloned into Ubi-TM vector that had been digested with ClaI and XhoI. Each clone was fully sequenced to confirm that no additional mutations were induced.

E15 primary mouse cortical neurons were cultured in 12-well plates as previously described [17], [18]. Neurons were co-transfected with GFP and empty plasmid or shank3 WT, Y1015X, or A921T plasmids using lipofectamine 2000 (Invitrogen) at 4 days *in vitro* (DIV). Cells were fixed in 4% paraformaldehyde for 20 min at room temperature. An anti-GFP rabbit IgG pool (Invitrogen) and a secondary anti-rabbit IgG antibody were applied, and the cells were visualized by Nikon fluorescence microscopy.

### Quantitative measurements of neurite outgrowth

Transfected neurons were selected at random from three independent transfections for each construct (20–25 cells per construct). Image J software from the National Institutes of Health (NIH) was used to convert 16-bit images to 8-bit gray-scale images. Neuronal morphology was traced using the Neurolucida v9.0 system (MicroBrightField, Williston, VT). The number of neurite, neurite nodes (branch points), neurite tips (terminals) and the cumulative lengths of traced neurons were calculated using NeuroExplorer (MicroBrightField, Williston, VT). Statistical analysis was performed using a one-way ANOVA and a Post Hoc test in SPSS10.0. The significance level was set at P<0.05.

## Results

### Patients with 22q13 deletions

**Figure 1:**
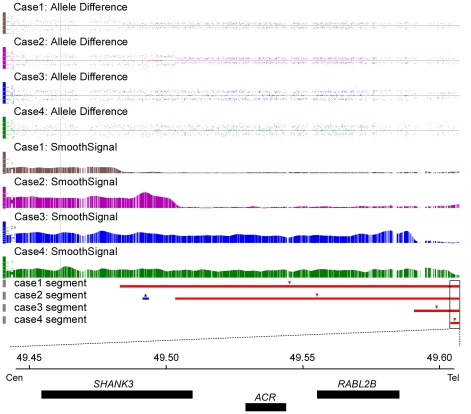
High-density SNP-array Analysis of Chromosome 22q13 Deletions in Four Patients with ID. Microdeletions and duplications are represented by red and blue bars respectively. Annotated genes in the overlapping region are shown in the bottom of the figure.

Four unrelated individuals with ID were found to have deletions of 22q13 ranging in size from 113 Kb to 4.4 Mb. All 4 cases shared a refined 113 Kb deletion that encompassed the gene *SHANK3* (Figure 1). These CNVs were identified by all four CNV-calling algorithms, and we designed a MLPA assay as an independent method to verify that all 22q13 deletions were heterozygous. Blood samples were obtained from both parents in cases 1 and 3 and one parent each for cases 2 and 4 to perform a parental study of the 22q13 deletions by MLPA. The results revealed that none of the parents carried the 22q13 imbalance. The basic features of each of the 4 cases are summarized in Table 1.

**Table 1:** Basic features of 4 cases with 22q13 deletion.

Characteristics	Case 1	Case 2	Case 3	Case 4
Sex	M	M	M	M
Age	8Y	6Y9M	2Y	13Y
CNV				
Cytogenetic position	22q13.31-33	22q13.31-33	22q13.33	22q13.33
Physicial position	45193851–49581309	45908122–49581309	48993966–49581309	49468229–49581309
Del/dup	del	del	del	del
Size(Kb)	4387	3673	587	113
*De novo*/inherited	*de novo*	N^a^	*de novo*	N^a^
Affected genes	47	44	27	4
Developmental				
Mental retardation	+	+	+	+
Speech and language delay	+	+	+	+
Motor development delay	+	+	+	+
Autistic behaviour	+	+	−	−
Physical				
Dysmorphisms	−	−	−	−
Hypotonia	−	−	+	−
MRI	−	+	N	N
Family history	+	−	−	−

a N:just one parent of the case was tested with MLPA and did not carry the 22q13 deletion.

Case 1 (8 years old) was the only child of non-consanguineous parents. He was born at 40 weeks after an uncomplicated vaginal delivery with a normal birth weight (3 kg). He had severe ID with an IQ of 30. He could not walk until 2 years of age and could not use words at the time he was recruited into the study at 8 years of age. The patient made no eye contact and no response to calling and presented impaired social communication, restricted and stereotyped patterns of behaviors and interests, and aggressive behaviors. Physical examination identified a relatively small head circumference. An MRI of the brain revealed no abnormalities. His father exhibited mild stuttering, and his aunt had a mild psychiatric abnormality that had not been clinically diagnosed. Case 2 (6 years, and 9 months old) was the only child of non-consanguineous parents. He had ID with an IQ<50. He showed delayed motor development and prominent speech and language developmental delays. At the age of 6 years and 9 months, he could walk, speak simple words, and implement simple commands. Additionally, he exhibited autistic behaviors such as poor eye contact and restricted interests. Physical examination identified one 9×6 mm café-au-lait spot on the skin. An MRI of the brain revealed long T1 and T2 signals at the posterior horn of the bilateral ventricles and the periventricle. Case 3 (2 years old) was the only child of non-consanguineous parents. He was affected with a severe developmental delay and hypotonia with an IQ<50. When he was referred at the age of 2 years, he could not walk or use words. There were no notable events in his family history. Case 4 (13 years old) was the only child of non-consanguineous parents. He exhibited delayed speech and motor development with an IQ<50. He could not walk or speak simple words until the age of 3 years. Physical examination revealed no abnormalities.

In summary, all cases presented with moderate to severe ID, pronounced delays in speech and language development, and abnormal motor development.

### Low frequency of 22q13 deletion in the literature

In all, 21,281 cases from 29 studies were included for our analysis of the literature (Table S1). Of 21,281 cases, the majority was from Europe, America and Australia. Fifty-two cases with ID, DF or MCA (0.24%) were identified to have the 22q13 deletion. Compared with this result, the proportion of 22q13 deletions involving *SHANK3* in our Chinese cohort (1.7%, 4/230) was much higher.

### Mutation detection of *SHANK3* and in vitro functional analysis

**Figure 2:**
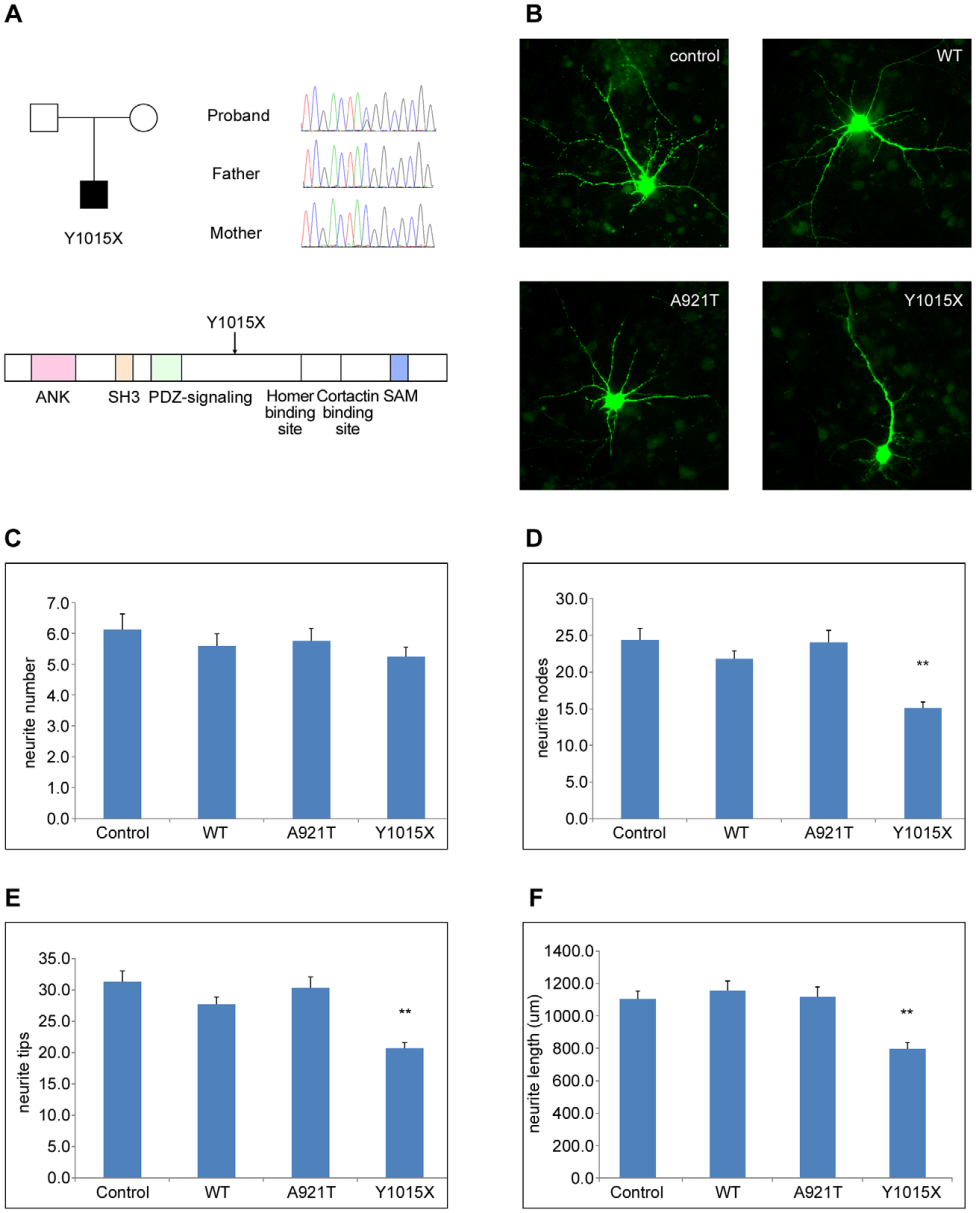
Identification and functional analysis of *SHANK3* mutations. (A) Pedigree of the proband with a *de novo* Y1015X mutation and localization of this nonsense variation on the linear protein structure of *SHANK3*. ANK, ankyrin repeats; SH3, Src homology 3 domain; PDZ, postsynaptic density protein; SAM, sterile α motif domain. (B) Representative photographs demonstrating the anatomic differences in traced neurons transfected with empty vector, *SHANK3* WT, A921T, or Y1015X vectors, respectively. (C–F) Analysis of *SHANK3* mutations in primary mouse cortical neuronal cultures. Neurite number (C), nodes (D), tips (E) and total length (F) are quantified in bar histograms along with standard error of the mean for each bar. Asterisks indicate a statistically significant difference (**P<0.001, Post Hoc tests). Compared to control, WT and A921T, the overexpression of Y1015X significantly decreased neurite complexity and length.

We screened the coding region and exon-intron splice junctions of the *SHANK3* gene in 234 unrelated ID patients. An unreported heterozygous nonsense mutation (Y1015X) was identified in one case, and absent in the parents and 556 controls (Figure 2). The boy has ID and epilepsy. The Y1015X mutation of the *SHANK3* gene lead to a predicted truncated protein of 1,015 amino acid residues compared with WT SHANK3 (1,747 amino acid). Additionally, in another single case, we identified a missense mutation (A921T) that was inherited from the patient's mother and absent in 556 controls. The boy was diagnosed with ID only. The affected boy showed motor and intelligence developmental delays. He could walk at the age of 26 months and speak simple words at the age of three and half years. There was no positive history of birth and family. Physical examination and MRI were negative.

To determine whether *SHANK3* mutations affect neuronal morphology, we transfected primary mouse cortical neurons with an empty vector or a vector encoding WT, A921T or Y1015X and traced the neurons. Post Hoc tests showed that neurons transfected with Y1015X had significantly decreased numbers of neurite nodes and tips and decreased cumulative length compared with neurons transfected with control, WT, or A921T (P<0.001, Figure 2), while no significant difference in neurite number was observed. No statistically phenotypic differences were observed between A921T and WT. These results suggest that the *SHANK3* Y1015X mutation plays an important role in neurite development, while the A921T mutation is unlikely the causal mutation.

## Discussion

Since the first case of 22q13 deletion syndrome was reported by Watt in 1985 [19], the case reports have accumulated rapidly [20], [21]. However, the diagnosis of this disorder remains difficult in the clinic because the features are non-specific and the experimental testing is largely dependent on the resolution of the methods available. More than ten different methods are used to test for subtelomeric imbalances, with a variable resolution from several Mb to 100 Kb or less. The known smallest deletion of 95 Kb was identified using the Agilent array-CGH 244K method by Dhar et al. [22]. In the present study, the high-resolution platform of the Affymetrix Human SNP 6.0 array was used to test for CNVs in the Chinese population. Four out of 230 cases (1.7%) were identified to carry deletions in 22q13 ranging in size from 113 Kb to 4.4 Mb.

We reviewed 29 genome-wide microarray studies of ID, DF or MCA and identified 52 of 21,281 cases (0.24%) with 22q13 deletions. Compared with this data, the frequency of 1.7% for 22q13 deletion syndrome in our ID cohort is significantly higher, supporting the widely held speculation that the frequency of 22q13 deletion has be underestimated. The possibility of false positive cases was excluded since four different software packages were used to detect the CNVs and the positive cases were validated by MLPA. The discrepancy of the frequency could be explained by the complexity of sample phenotypes, heterogeneity of genetic factors and various experimental methods. During the literature review, we included the studies based on the criteria that the majority of the cases in each report were affected with ID, but the situation of co-morbidity and the ascertainment methods were various among different studies. The phenotypic complexity of the samples could result from the heterogeneity of genetic factors. BAC arrays or low-resolution microarrays were used to detect CNVs in most studies reviewed. In this study, we screened all segments longer than 50 Kb for CNVs using Affymetrix Human SNP 6.0 array. Compared to conventional karyotyping and low-resolution microarrays, the high-resolution microarray methods such as SNP 6.0 could cut down the false negative rates. Affymetrix Human SNP 6.0 array could detect smaller CNVs<500 Kb, even<100 Kb. The 29 genome-wide microarray ID studies reviewed in the literature were performed in primarily Caucasian populations, while the subjects in this study were exclusively Chinese. Jiang et al. screened 451 Chinese ID cases for CNV detection using subtelomere-MLPA method. Two deletions and one duplication in 22q13 were identified, accounting for 0.67% of cases.[15] We used the same ethnic population screened by the same including and excluding criteria, but compared with Jiang's study, we used different methods to access the frequency of 22q13 CNVs in ID. The Affymetrix Human SNP 6.0 array we used has detailed probes with an average spatial resolution of 700 bp, while subtelomere-MLPA contains only a few probes for a certain region of chromosomes, which is expected to have low detection rate. Our results suggest that the frequency of 22q13 deletions is unexpected high in Chinese ID patients. A limitation of this study was the relatively small sample size (230 cases versus 101 controls). For this reason, more samples from Chinese population should be studied to validate these preliminary results.

It should be mentioned that we found one female DD case carrying a 22q13 duplication of 5.86 Mb, spanning 22q13.31 to 22qter. However, a detailed clinical phenotype was not available as this subject died at the age of 2 years and therefore was not included in our analysis.

The smallest CNV in 22q13 that we identified spanned 113 Kb and affected the gene *SHANK3*. One *de novo* nonsense mutation (Y1015X) in *SHANK3* was identified in ID. The mutation of tyrosine substituted by a stop codon at amino acid position 1015 (Y1015X) leads to a predicted truncated protein lacking the Homer- and Cortactin-binding sites as well as the sterile α motif (SAM) domain. The mouse cortical neurons transfected with Y1015X construct showed dramatically decreased numbers of neurite tips and nodes and the cumulative length compared with control, WT and A921T. We also identified a missense mutation A921T in an ID patient, inherited from his unaffected mother. Functional analysis did not find any influence of this mutation on neurite development. Both the inheritable transmission pattern and non-functional effect indicated that mutation A921T might be benign.

Our results showed that SHANK3 protein is important for neurogenesis, consistent with other reports [23], [24], [25], [26]. Effects of SHANK3 on dendritic spine synapses in neurons were demonstrated by the fact that the knock-down of Shank3 by RNA interference in hippocampal neurons reduces spine density, whereas transfection of this protein in aspiny neurons induces formation of new functional synapses [23]. The study of Shank3 heterozygous mice also found that haploinsufficiency of Shank3 resulted in a decrease in synaptic transmission, altered functional and structural plasticity of synapses and reduced social behaviors [24]. Meanwhile, mice with homozygous Shank3 gene deletions displayed morphological defects of spiny neurons, including neuronal hypertrophy and a significant reduction in spine density [25]. Shank3 was further found to be at the tip of actin filaments and participates in growth cone motility in developing neurons. SHANK3 mutations identified in autism affect the development and morphology of dendritic spines via an actin-dependent mechanism [26].

*SHANK3* is a binding partner of neuroligin, which is expressed in postsynaptic neurons and interacts with presynaptic neurexins. Neuroligins-Neurexins pathways are believed to play an important role in the pathogenesis of neurodevelopmental disorders. Mutations in *SHANK3* have been reported in individuals with mental retardation, autism, and pervasive developmental delays [27]. Interestingly, Gauthier et al. sequenced the *SHANK3* gene in 185 schizophrenia patients and detected a *de novo* nonsense R1117X mutation in one patient and his two affected brothers [28]. Though the gene *SHANK3* is well-known susceptibility gene for neurodevelopmental disorders, there was only one report about the mutation screening of this gene in ID [14]. Our report was the second one about the mutation screening of *SHANK3* gene in ID and sample size was larger than the first one (95 individuals). These evidences suggest that phenotypic variations as a result of *SHANK3* mutations are quite broad and that ID, ASDs and schizophrenia may share common etiological factors.

In conclusion, this study indicates that frequency of 22q13 deletion is as high as 1.7% in Chinese ID population. The observation of frequent 22q13 deletions and a mutation that led to impaired function of SHANK3 in ID patients supports the haploinsufficiency role in etiology of ID.

## Supporting Information

Table S1 Overview of 22q13 deletions detected by genome-wide microarrays in ID.
(DOC)
