# Supplementary material for: High Proportion of 22q13 Deletions and SHANK3 Mutations in Chinese Patients with Intellectual Disability
Source: PLoS One. 2012 Apr 11;7(4):e34739. doi: 10.1371/journal.pone.0034739 (PMC3324537; doi:10.1371/journal.pone.0034739)
Supplement: Table S1 — Overview of 22q13 deletions detected by genome-wide microarrays in ID. (DOC) [file pone.0034739.s001.doc]

**Table S1: Overview of 22q13 deletion**s detected by genome-wide microarrays in ID

| **Author(year)** | **Country** | **No. of patients** | **Phenotype** | **Array platform** | **Number of 22q13 del** | **Frequency (%)** |
| --- | --- | --- | --- | --- | --- | --- |
| Vissers et al.  (2003) | Netherlands, U.S. | 20 | MR, DF | BAC | 0 | 0 |
| Shaw-smith et al. (2004) | UK., France | 50 | MR | BAC | 0 | 0 |
| De Vries et al.  (2005) | Netherlands | 100 | MR | BAC | 0 | 00 |
| Schoumans et al. (2005) | Sweden | 41 | MR | BAC | 0 | 0 |
| Tyson et al.  (2005) | Canada | 22 | MR, DF | BAC | 0 | 0 |
| Friedman et al.  (2006) | Canada | 100 | MR | Affy 100K | 0 | 0 |
| Krepischi-Santos et al. (2006) | Brazil | 95 | MR | BAC | 0 | 0 |
| Menten et al.  (2006) | Belgium | 140 | MR, MCA | BAC | 1 | 0.7 |
| Miyake et al.  (2006) | Japan | 30 | MR | BAC/PAC | 1 | 3.3 |
| Rosenberg et al.  (2006) | Netherlands, Brazil, UK | 81 | MR, DF | BAC | 0 | 0 |
| Aradhya et al.  (2007) | U.S. | 20 | DD, MR, MCA | Agilent 44k | 0 | 0 |
| Engels et al.  (2007) | Germany | 60 | MR | BAC | 0 | 0 |
| Fan et al.  (2007) | U.S. | 100 | MR | Agilent 44k | 0 | 0 |
| Hoyer et al.  (2007) | Germany | 104 | MR | Affy 100K | 0 | 0 |
| Shen et al.  (2007) | U.S. | 211 | MR, DD | Agilent | 0 | 0 |
| Thuresson et al.  (2007) | Sweden | 48 | MR | BAC | 0 | 0 |
| Wagenstaller et al. (2007) | Germany | 67 | MR | Affy 100K | 0 | 0 |
| Baldwin et al.  (2008) | U.S. | 211 | DD,MR,DF,CA,ASD | Agilent CGH | 0 | 0 |
| Nowakowska et al. (2008) | U.S. | 116 | MR | BAC, CGH | 0 | 0 |
| Pickering et al.  (2008) | U.S. | 1176 | MR,DD | BAC | 3 | 0.26 |
| Xiang et al.  (2008) | U.S. | 50 | DD,MR | Agilent 44K | 0 | 0 |
| Bruno et al.  (2009) | Australia | 117 | MR | Affy 250K | 0 | 0 |
| Friedman et al.  (2009) | Canada | 100 | MR | Affy 500K | 0 | 0 |
| Gijsbers et al.  (2009) | Netherlands | 318 | MR,MCA | Affy 250K, Illumina 300 | 0 | 0 |
| Koolen et al.  (2009) | Netherlands | 386 | MR, CA, DF | BAC | 0 | 0 |
| McMullan et al.  (2009) | Netherlands, Germany, UK | 120 | MR | Affy 500K | 0 | 0 |
| Xiang et al.  (2010) | U.S. | 1499 | MR, DD | Agilent 44K | 2 | 0.13 |
| Jaillard et al.  (2010) | France | 132 | MR | Agilent 44K | 0 | 0 |
| Cooper et al.  (2011) | U.S. | 15767 | MR, DD, CA | BAC, oligo-based chip (105K or 135K) | 45 | 0.29 |
| Total |  | 21281 |  |  | 52 | 0.24 |
